# Structural Similarity and Classification of Protein Interaction Interfaces

**DOI:** 10.1371/journal.pone.0019554

**Published:** 2011-05-12

**Authors:** Nan Zhao, Bin Pang, Chi-Ren Shyu, Dmitry Korkin

**Affiliations:** 1 Informatics Institute and Department of Computer Science, University of Missouri, Columbia, Missouri, United States of America; 2 Bond Life Science Center, University of Missouri, Columbia, Missouri, United States of America; University of South Florida College of Medicine, United States of America

## Abstract

Interactions between proteins play a key role in many cellular processes. Studying protein-protein interactions that share similar interaction interfaces may shed light on their evolution and could be helpful in elucidating the mechanisms behind stability and dynamics of the protein complexes. When two complexes share structurally similar subunits, the similarity of the interaction interfaces can be found through a structural superposition of the subunits. However, an accurate detection of similarity between the protein complexes containing subunits of unrelated structure remains an open problem.

Here, we present an alignment-free machine learning approach to measure interface similarity. The approach relies on the feature-based representation of protein interfaces and does not depend on the superposition of the interacting subunit pairs. Specifically, we develop an SVM classifier of similar and dissimilar interfaces and derive a feature-based interface similarity measure. Next, the similarity measure is applied to a set of 2,806×2,806 binary complex pairs to build a hierarchical classification of protein-protein interactions. Finally, we explore case studies of similar interfaces from each level of the hierarchy, considering cases when the subunits forming interactions are either homologous or structurally unrelated. The analysis has suggested that the positions of charged residues in the homologous interfaces are not necessarily conserved and may exhibit more complex conservation patterns.

## Introduction

Interactions between proteins form protein complexes and underlie many cellular processes [1]. When studying evolution of protein interactions or predicting and structurally characterizing new interaction interfaces, the concept of interaction similarity often plays a principal role [2], [3], [4]. The properties of similar interfaces have been analyzed on a large scale by a number of research groups. For instance, it has been shown that the geometry of interactions is often conserved between similar pairs of proteins [2]. Another study has revealed that homologous proteins often have their binding sites in similar locations on protein surfaces to interact with other, sometimes unrelated, proteins [4]. While similarity of the interfaces in homologous protein complexes is not surprising, it is not clear to what extent two structurally unrelated complexes can have similar, “analogous”, interfaces. Recently, a new phenomenon of molecular mimicry in host-pathogen interactions has been reported, where a pathogenic protein acquires a binding surface similar to that of a host protein, presumably through convergent evolution [5], [6], [7], [8]. As a result, the pathogenic protein competitively binds to another host protein, forming an analogous interface, similar to the interface between the two host proteins, and thus hijacking an important cellular function. The available experimental data suggest that pathogenic agents extensively use the molecular mimicry to their advantage [6]. Molecular mimicry can also occur in the intra-species interactions [9]. Studying analogous interfaces is challenging since it requires an accurate method to detect similarity between the interfaces of structurally unrelated protein-protein interactions.

Several approaches to quantify the interface similarity have been proposed to date. Some approaches rely on a superposition of the entire structures of the interacting proteins [10], [11]. For instance, this can be done by calculating the ligand root mean square deviation (*L_RMSD*) measure, which is defined as a RMSD value between the back-bones of the smaller subunits (ligands), once the corresponding larger subunits (receptors) are superimposed [12]. While such an approach can provide the most accurate estimation of the interaction similarity between the closely related complexes, it may not be applicable to the cases of distant homology between the protein complexes, or even convergent evolution, where an accurate superposition of subunits is not feasible. Another way to define the interaction similarity is through the similarity of the corresponding interaction interfaces. This can be done by using an RMSD measure calculated only for the superposed interface structures, while not taking into account the overall structures of the interacting subunits [3], [13], [14], [15]. The latter approach, while faster than the one using the whole-subunit superposition, could further benefit from additional information about the interacting residues.

Interaction similarity is also used to cluster protein-protein interactions [3], [14], [16], [17]. For instance, an interface prediction and classification system, Prism, defines structural similarity by aligning the binding sites that form each interface using MultiProt software [18]. In total, there are 21,684 interfaces collected in Prism, which are clustered into 3,799 clusters based on their structural similarity. Another classification system, SCOPPI, uses a two-stage classification system to cluster binding sites within each SCOP family [16]. In the first stage, the binding sites are clustered based on a sequence pattern of their contact residues. In the second stage, the initial groups of binding sites are merged into the larger clusters, based on the similarity of geometrical features of the binding sites. The interfaces can then be clustered, based on the clustering of their binding sites. While classification of protein interactions of homologous subunits has been addressed by several approaches, an accurate classification of analogous interfaces remains a challenge.

The goal of this work is finding an accurate alignment-free interface similarity measure and demonstrating its advantages and applicability. First, we introduce an accurate structure-based interface similarity measure that is used to generate a training set of similar and dissimilar interfaces. We then describe a feature-based interface similarity measure by employing a supervised learning approach, which is trained on the known structures of protein-protein interfaces. Furthermore, we apply the feature-based similarity measure to develop (i) a proof-of-concept hierarchical classification of protein interactions, and (ii) a data structure for efficient search and retrieval of similar interfaces. The classification can be useful in the evolutionary studies of protein interactions, as illustrated by our case study analysis.

## Methods

Our methods are organized as follows. First, we define and compare two structure-based interface similarity measures, *iiRMSD* and *siRMSD*. Second, we apply the more accurate of the two measures to a non-redundant set of protein interfaces to determine reliable positive and negative training sets for our feature-based measure. Third, we use the training set to obtain two Support Vector Machine (SVM) models, resulting in two feature-based interface similarity measures. Finally, we employ one of the new feature-based similarity measures to (i) define a structure-based hierarchical classification of protein interaction interfaces on a large scale, and (ii) design a data structure for the interface search and retrieval problem.

### Basic concepts: Homology and analogy in protein-protein interactions

We first formally define the concepts of a *protein-protein interaction*, *protein binding site*, and *protein interaction interface* since these concepts will be used throughout the paper. A protein-protein interaction is defined as a triple (*S_1_*, *S_2_*, *O*), where *S*_1_ and *S*_2_ are the two interacting subunits (either proteins or protein domains), and *O* is their relative orientation. A residue *r_1_* of one subunit is in contact with residue *r*_2_ of another subunit if *r*_1_ has at least one atom within 6 Å of an atom of *r*_2_. The set of all residues from one subunit that are in contact with any residues of another subunit constitutes a protein binding site. For a protein-protein interaction, its interaction interface is defined by a triple (*B*_1_, *B*_2_, *C*), where *B*_1_ and *B*_2_ are the binding sites of the interacting subunits, and *C* is a set of all pairs of residues that are in contact.

We next introduce three types of similar interaction interfaces based on the protein-protein interactions they mediate. Two protein-protein interactions that share similar interfaces are called *homologous* if a subunit in the first interaction shares homology with a subunit in the second interaction, and the remaining two subunits also share homology between each other. Two protein-protein interactions that share similar interfaces are called *common-partner analogous* if a subunit in the first interaction shares homology with a subunit in the second interaction, while the remaining two subunits are structurally unrelated. Finally, two protein-protein interactions that share similar interfaces are called *analogous* if both subunits in the first interaction are structurally unrelated to subunits in the second interaction. The protein interfaces formed by interactions of the three types are called homologous, common-partner analogous, and analogous, correspondingly.

### Comparing two structure-based similarity measures

**Figure 1:**
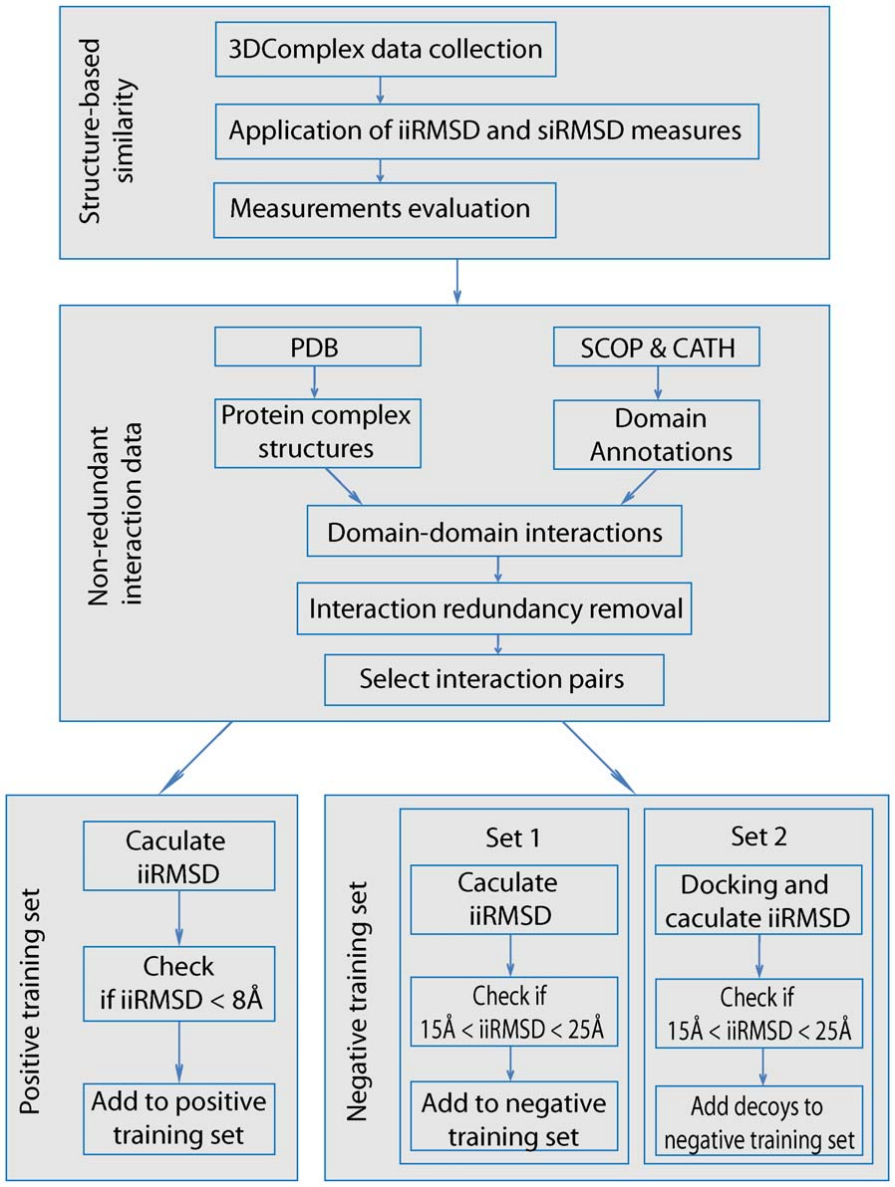
A protocol for obtaining a reliable set of similar and dissimilar interface pairs. First, two structure-based similarity measures, *iiRMSD* and *siRMSD*, are evaluated on a dataset collected from 3D Complex database. Second, a non-redundant domain-domain interaction data set is obtained from PDB, SCOP and CATH. Third, *iiRMSD* is used to classify positive (similar) and negative (dissimilar) training sets of pairs of interaction interface structures.

To train a feature-based similarity measure, one needs to generate two reliable training sets of similar (positive training set) and dissimilar (negative training set) interfaces. This is done by employing a structure-based similarity measure, a commonly used approach to compare homologous interfaces or interfaces formed by the same subunits [12]. The set-generating protocol consists of three stages (Fig. 1). First, we define two structure-based interface similarity measures: one that relies on structural superposition of the entire protein complexes and another one that relies on superposition of the protein interfaces. Second, we prepare a candidate dataset of pairs of non-redundant protein-protein interactions, where each participating subunit is classified based on its evolutionary relationships to other subunits. Third, we compare the structure-based similarity measures and apply the most accurate measure to the candidate dataset to determine a positive training set that includes homologous and common-partner analogous pairs of interfaces and a negative training set of structurally unrelated interfaces.

The first structure-based similarity measure, the interaction interface RMSD (*iiRMSD*), is defined by superposing overall structures of the interacting subunits, similar to *L_RMSD* measure, used in CAPRI docking assessment [12]. Given two protein-protein interactions, one between subunits *A*_1_ and *A*_2_ and another one between subunits *B*_1_ and *B*_2_, we calculate *iiRMSD* through the following steps:

1. Structurally align subunit *A*_i_ with another subunit *B*_j_ (*i*,*j* = 1,2) using MultiProt software [18]; calculate C_α_-only RMSD between the corresponding residues of the binding sites of *A_i_* and *B_j_*

2. For each alignment *A_i_*−*B_j_*:

2.1 Superpose the remaining two subunits according to the alignment; and calculate C_α_-only RMSD between the corresponding residues of the binding sites of remaining subunits

2.2 Calculate an average of the two C_α_-only RMSD values

3. Select the smallest of the calculated averages over four possible superposition scenarios for *A_i_* and *B_j_*.

The second similarity measure, the superposed interface RMSD (*siRMSD*), is defined as the C_α_-based RMSD between the corresponding residues of the structurally superposed interaction interfaces. The structural superposition of interfaces is done using the same MultiProt software [18]. Thus, in contrast to *iiRMSD*, *siRMSD* is guided exclusively by the local structure of the interaction interfaces, which can potentially lead to the incorrect detection of similar interfaces, specifically when the interface structures are small.

Next, we compare accuracies of both measures by applying them to a dataset of homologous and dissimilar protein interfaces extracted from 3D Complex, a non-redundant database of protein complexes that are classified based on their similarity in sequence, structure, or topology [19]. The hierarchical classification system in 3D Complex consists of 12 levels: protein complexes of different topologies are separated at the first level, while complexes of the same topology and geometry but with varied sequence identities are separated at one of the last 8 levels (Levels 4–12). In this work, the pairs of complexes were selected from the third, Quaternary Structures (QS), level. At this level, protein complexes grouped in the same cluster have the same topology, domain architecture, and stoichiometry, as well as share the evolutionarily related proteins.

Our simple assumption behind extracting similar interaction interfaces from 3D Complex is that two structurally similar protein complexes are likely to have structurally similar interaction interfaces. First, 5,924 pairs of structurally similar complexes are selected from 4,005 clusters of protein complexes at the QS level of 3Dcomplex. We randomly select two complexes from each cluster if it has more than one protein complex. It is not difficult to see that all collected pairs of similar interfaces satisfy our definition of homologous interfaces. Second, we generate a set of 4,491 pairs of structurally unrelated protein complexes. To do so, pairs of complexes are randomly selected from different clusters, such that the pairs of binary interactions extracted from these complexes are formed by four different subunits (i.e., different homologous chain IDs for all four subunits). To exclude a rare possibility of different binding modes that can occur for a pair of homologous or even identical proteins, all pairs of obtained proteins are manually checked using subunit sequence similarity and symmetry information from 3D Complex.

Finally, *iiRMSD* and *siRMSD* measures are calculated and compared for all similar and dissimilar interface pairs in the dataset. Specifically, we use Bhattacharyya Coefficient based metric [20] to compare the distributions of similarity values between the sets of similar and dissimilar interfaces generated by each measure. Based on evaluation of the histograms obtained from *iiRMSD* and *siRMSD* similarity distribution, using *n* = 50 bins (see section *Comparison of structure-based interface similarity measures* in *Results*), *iiRMSD* is selected to obtain the set of similar and dissimilar protein interfaces.

### Obtaining training sets of similar and dissimilar protein-protein interfaces

To obtain reliable training sets of interaction interfaces, we calculate the *iiRMSD* values between the pairs of interfaces extracted from a diverse non-redundant set of protein-protein interactions. First, the protein-protein interactions are collected from PIBASE, a database of protein interaction structures [21]. Second, we remove the interaction structures with resolution worse than 2.5 Å (the resolution is obtained from the protein Data Bank, PDB [22]) and interactions formed by redundant subunits. We define redundant subunits as the structures that share at least 95% sequence identity, using ASTRAL SCOP 1.75 [23]. In total 1,383 non-redundant binary protein interactions are extracted from the high-resolution structures. Third, each of the two subunits in a protein-protein interaction is assigned a SCOP Superfamily ID [24]. Proteins from the same SCOP Superfamily are evolutionary related, based on structural, functional, and sequence evidence. Fourth, all interactions are grouped based on their SCOP Superfamily IDs such that interactions within the same group share the same pairs of assigned SCOP Superfamily IDs. Finally, we consider only those groups that have two or more interactions, resulting in 585 groups of 2,296 interfaces in total.

As mentioned before, our positive training set of similar interfaces includes homologous and common-partner analogous interfaces. Ideally, one would like to have a positive set that includes all three types of similar interfaces: homologous, common-partner analogous, and analogous. However, it is not feasible to generate a reliable set of analogous interfaces using *iiRMSD* or any other similarity measure that relies on subunit superposition since it may not be possible to structurally align the pairs of interacting subunits. While it may be feasible to implement the definition of the analogous interface using a similarity measure that relies solely on the interface superposition such as *siRMSD*, selecting a reliable set of analogous interfaces for the positive set using such method remains a problem.

To obtain the set of homologous interfaces, we consider all possible non-redundant interface pairs within the same SCOP Superfamily group of interfaces. In total, we have considered 7,206 interface pairs. Then, we select a pair of interfaces as similar interfaces if the *iiRMSD* measure between them is smaller than 8 Å. This threshold was selected to minimize the number of false-positives, based on our analysis of *iiRMSD* values for similar and dissimilar interfaces (see section Comparison of structure-based interface similarity measures in Results). As the result, we obtained 372 pairs of homologous interaction interfaces (Table 1). We will refer to these data as *Positive_H_*. To obtain the set of common-partner analogous interfaces, we first determine all pairs of interfaces that share a common SCOP Superfamily for exactly one subunit in each interface. In total, 14,509 pairs of interface SCOP Superfamily groups containing 29,180 interface pairs were selected. For each interface pair we calculate the *iiRMSD* measure, which requires superposition of only one pair of subunits and therefore can be applied to a pair of interfaces with other two subunits being structurally unrelated. We then use the same upper bound of 8 Å to define similar interfaces, resulting in 480 pairs of common-partner analogous interfaces. We will refer to these data as *Positive_C_*.

**Table 1:** Positive and negative datasets.

Dataset	Subsets	N_IP_	Total	Threshold
Positive set	*Positive_H_*	372	852	*iiRMSD*<8 Å
	*Positive_C_*	480		
Negative set	*Negative_NN_*	723	1322	15 Å<*iiRMSD* and *iiRMSD*<25 Å
	*Negative_ND_*	599		

*N_IP_* is the number of interface pairs from each subset of the positive and negative datasets after the RMSD thresholds are applied. *Total* is the number of pairs in each dataset. *iiRMSD* is used to define an upper threshold for the positive set (8 Å) as well as the lower and upper thresholds for the negative set (15 Å and 25 Å). The thresholds are imposed to minimize the number of false positives and negatives.

To obtain a negative set of dissimilar interface pairs, two strategies are considered. In the first strategy, we compare a ‘native’ interface from the dataset of non-redundant interactions, described earlier, with a ‘decoy’ interface formed using the same subunits. The subunits are first detached and then re-docked by a protein docking method. In the second strategy, we compare a pair of native interfaces. Specifically, in the first strategy we randomly select 4,309 native interfaces; for each pair of subunits forming an interface, a set of 4,309 decoy interfaces is then obtained by detaching the subunits followed by their re-docking using PatchDock software [25]. The *iiRMSD* measure is then calculated between the native interface and each of the decoy interfaces; the lower and upper threshold of 15 Å and 25 Å, respectively, are used to select the final set of dissimilar interface pairs. The lower threshold is selected based on the evaluation of *iiRMSD* measure. The upper threshold is used to exclude extreme dissimilarities that are due to any significant errors in alignments and can reduce the sensitivity of our SVM classifiers. In total, 599 dissimilar native-decoy interface pairs have been determined (Table 1). We will refer to these data as *Negative_ND_*.

In the second strategy, we determine the set of structurally unrelated interface pairs extracted exclusively from native structures by (1) randomly selecting a pair of interactions from the non-redundant set, such that all four subunits forming the interactions belong to four different SCOP Superfamilies, (2) determining the *iiRMSD* values between the interfaces, and (3) applying the same lower and upper thresholds (15 Å and 25 Å) as in the first strategy. As a result, 723 dissimilar native-native interface pairs were selected (Table 1). We will refer to these data as *Negative_NN_*.

### A machine learning approach to train a feature-based similarity measure

To determine whether two interaction interfaces are similar without the use of structural alignment, we train a feature-based similarity measure using a Support Vector Machines (SVM) approach [26]. SVMs have been successfully used in a number of bioinformatics applications [27], [28]. Given a positive training set of *n*_1_ pairs of similar and *n*_2_ pairs of dissimilar interfaces, where each pair is represented as a vector of *N* numerical features, ***x^i^*** = (*x*_1_,*x*_2_,…,*x_N_*), the basic goal is to train a classifier that would classify a pair of the interfaces as either similar or dissimilar. In its simplest form, the problem can be viewed as finding a hyperplane that separates two classes of points maximizing a margin defined by the closest to the hyperplane positive and negative examples. The formalism can be expanded by introducing non-linear classifiers defined through the kernel functions, For our approach we employ two widely used non-linear kernel functions: the polynomial kernel, 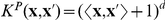, where *d* is degree of the polynomial, and the radial basis function (RBF), 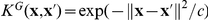. For both, SVM training and testing, we used *SVM_light_* software [29].

**Figure 2:**
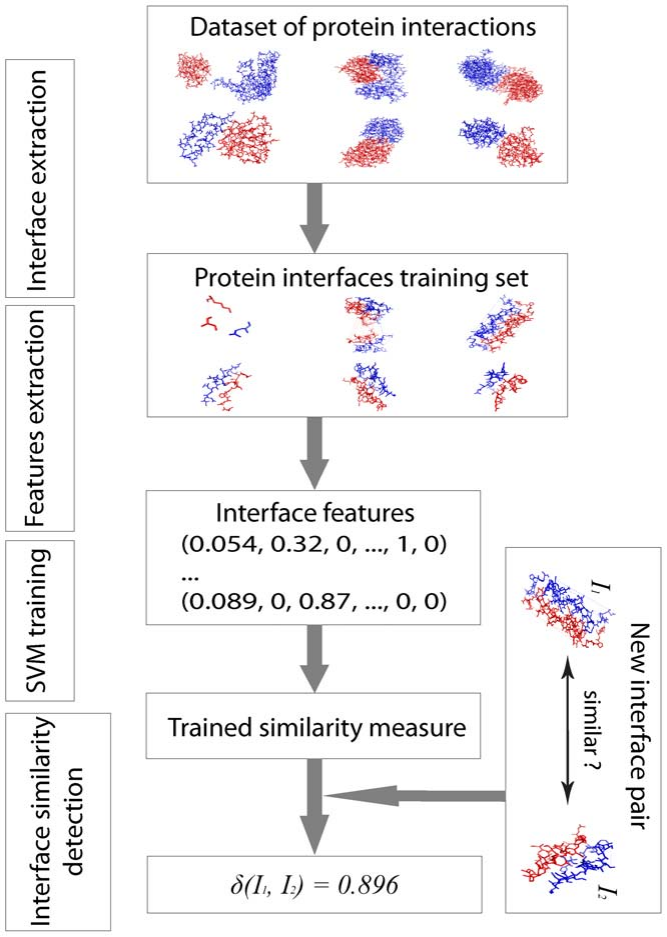
An overview of machine learning approach to determine interface similarity measure. First, interface structures are extracted from the training sets of similar and dissimilar interaction interfaces. Second, for each pair of interfaces a 106-dimensional feature vector is calculated. Third, a Support Vector Machines classifier is trained and evaluated using the above datasets. Last, a protein interface similarity measure *δ(I_1_, I_2_)* is defined for two interfaces, *I_1_* and *I_2_*, as the distance between the corresponding106-dimensional feature vector and the separating hyperplane.

Our approach consists of three main stages (Fig. 2). First, two datasets of interface pairs are extracted from our training sets. The first dataset includes a positive set of 852 interface pairs (372 from *Positive_H_* and 480 from *Positive_C_* sets), and a negative set of 599 pairs from *Negative_ND_* set. The second dataset includes the same positive set, but the negative set combines 723 interface pairs from *Negative_NN_* and 599 from *Negative_ND_* sets. Second, for each interface structure, we calculate a 53-dimensional vector, which consists of features describing geometrical and physico-chemical characteristics of the interfaces. For the training procedure, all interface feature vectors are paired up, resulting in 106-dimensional feature vectors. Third, two SVM classifiers are trained: classifier *Model_ND_* is based on the first dataset and classifier *Model_NDNN_* is based on the second dataset. Fourth, for each model, a protein interface similarity measure *δ(I_1_, I_2_)* is defined for two interfaces, *I_1_* and *I_2_* as the distance between the 106-dimensional feature vector and the separating hyperplane. We then convert the measure to a distance by subtracting each value from the observed maximum. Interestingly, when analyzing the converted similarity measure values calculated for the entire set of 2,806×2,806 interface pairs (which is later used for hierarchical classification; see next section *Structure classification of interaction interfaces* under *Methods*), we found that the measure obeys the triangle inequality rule. Finally, during the testing stage, we evaluate the accuracy of the feature-based similarity measures based on the two SVM models.

There are 5 different types of features that constitute each 53-dimensional feature vector. The first feature type is a one-dimensional feature defined as the difference between the numbers of contact residues in each interface. The second type represents statistics on the residue contact pairs between 7 basic residue groups based on the physico-chemical characteristics of the residues. The residue groups include aromatic, aliphatic, hydrophobic, small, negatively charged, positively charged, and polar residues, where each amino acid residue may belong to more than one group (Table 2) [30]. The occurrence frequency of a pair of contact residues in each pair of residue groups is calculated adding (7×8)/2 = 28 dimensions. The third feature type consists of 4 surface patch parameters [31]. These are interface solvent accessible surface area (ASA), protrusion, planarity, and hydrophobicity. The interface ASA is defined as the sum of two protein binding site ASAs, where each binding site ASA is calculated as an average of each contact residue ASA, calculated by NACCESS [32]. A protrusion index gives an absolute value for the extent to which a residue protrudes from the surface of a protein, and is defined as an average of the protrusion indices of each residue, computed using Protruder software [33]. The planarity of each interface is calculated by Surfnet, a software that evaluates the root mean square deviation (RMSD) of all interface atoms from the fitted least squares plane [34]. The hydrophobicity of each interface is defined as an average of the hydrophobicity values of each interface residue assigned using the hydrophobicity scale [35]. The last feature type is concerned with the hot spot residues in each interface. A *hot spot* residue in a protein interface is defined as a residue that makes significant contribution to the binding free energy. We use a computational alanine scanning approach to get all hot spot residues for an interface [36]. This feature type is calculated as a 20-dimensional vector, where the *i*-th coordinate of the vector corresponds to the occurrence frequency of the *i*-th residue type as a hot spot residue.

**Table 2:** Amino acid residue classes according to their physicochemical properties.

	Aliphatic	Aromatic	Positive	Negative	Small	Hydrophobic	Polar
ALA	0	0	0	0	1	1	0
ARG	0	0	1	0	0	0	1
ASN	0	0	0	0	1	0	1
ASP	0	0	0	1	1	0	1
GYS	0	0	0	0	1	1	0
GLU	0	0	0	1	0	0	1
GLN	0	0	0	0	0	0	1
GLY	0	0	0	0	1	1	0
HIS	0	1	1	0	0	1	1
ILE	1	0	0	0	0	1	0
LEU	1	0	0	0	0	1	0
LYS	0	0	1	0	0	1	1
MET	0	0	0	0	0	1	0
PHE	0	1	0	0	0	1	0
PRO	0	0	0	0	1	0	0
SER	0	0	0	0	1	0	1
THR	0	0	0	0	1	1	1
TRP	0	1	0	0	0	1	1
TYR	0	1	0	0	0	1	1
VAL	1	0	0	0	1	1	0

Six classes of residues were defined, where a residue may belong to more than one class.

The contribution of the individual features is analyzed using an SVM attribute evaluating protocol implemented in Weka [37]. This protocol is based on the SVM Recursive Feature Elimination method using weight magnitude as the ranking criterion [38]. To evaluate the obtained classification results for the two SVM models, we use a standard *leave-one-out* cross validation protocol for each SVM classifier [29]. The accuracy, *f_AC_*, is calculated as 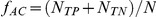, where *N_TP_* and *N_TN_* are the numbers of true positives and negatives, and *N* is the number of classified interfaces. The precision, *f_PR_*, is calculated as 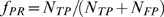 and the recall, *f_RE_*, is calculated as 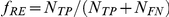.

### Structure classification of interaction interfaces

**Figure 3:**
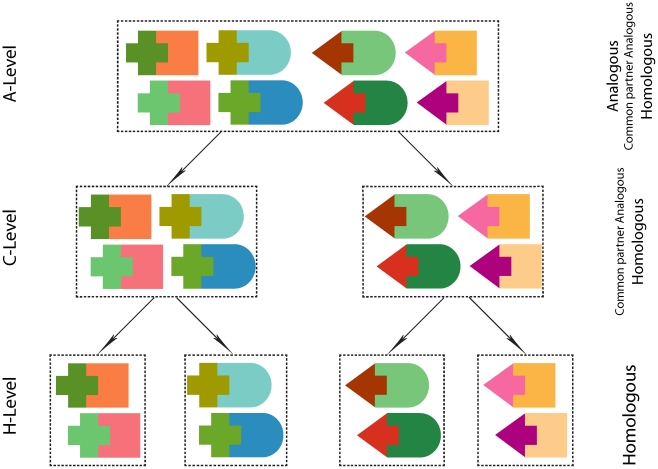
Hierarchical classification of interaction interfaces. Similar shapes correspond to homologous proteins. Three levels of structurally similar interaction interfaces are defined. A single cluster at H-level, C-level, and A-level can include homologous, common partner analogous, and analogous interfaces, correspondingly.

Using the new feature-based interface similarity, we develop a hierarchical classification of protein interfaces and applied it to a set of 2,806×2,806 interface pairs. The 2,806 interfaces are randomly sampled from our non-redundant set described in the previous section; they constitute ∼1% of all structurally determined interfaces [39]. The sampling procedure has been shown to reflect the distribution of similar interfaces among different SCOP Superfamilies. We use *Model_NDNN_*, as it has the higher accuracy in classifying dissimilar native interfaces (see section *Assessment of the new feature-based interface similarity measure* under *Results*). The hierarchy consists of three levels (Fig. 3), and is inspired by the classifications of protein structures, such as SCOP and CATH [16], [40]. At the first level, *A-level*, any two interactions from the same class can be analogous, common-partner analogous, or homologous. At the second level, *C-level*, two interactions from the same class can be either common-partner analogous or homologous. At the last level, *H-level*, only homologous interactions are allowed to be in the same class.

The hierarchy is obtained by first applying a similarity-based clustering procedure using the similarity measure derived from *Model_NDNN_* and then by imposing on each cluster the definitions of the three levels, starting from A-level and ending with H-level. To cluster interfaces, we used the *K*-medoid clustering method [41] on the whole data set of 7,873,636 interface pairs. *K*-medoid clustering is a generalization of *K*-means clustering not requiring for the similarity measure to satisfy the triangle inequality. To find an optimal threshold on the number of clusters, we use the Silhouette method, which compares the tightness and separation of clusters [42]. Each obtained cluster corresponds to an A-level class, as all interface pairs are similar to each other, while the interacting subunits may or may not be homologous (Fig. 3). Each A-level class is further split into one or more C-level classes by comparing the SCOP Superfamily IDs of all interacting proteins within the A-level class. Specifically, all interfaces whose subunits share at least one SCOP Superfamily ID in common are grouped into the same C-level class. Each C-level class is further split into one or more H-level classes; interfaces with both subunits sharing the same two SCOP Superfamily IDs are grouped in the same H-level class.

### Similarity-based retrieval of interaction interfaces

The above 3-level hierarchical clustering can be useful for studying the evolutionary and functional relationships between the protein-protein interactions with similar interfaces. However, it is likely to be inefficient for the interface retrieval problem, which asks: Given a query protein interface, how can one accurately and efficiently find a similar protein interface in a large interface dataset? Solving this problem requires development of a system for large-scale data organization, search and retrieval. In this section, we present an approach to index a protein interface database and make it searchable using an M-Tree [43]. The designed M-Tree is a data structure that relies on the feature-based representation of the interfaces. Specifically, we construct M-Tree in a top-down manner starting with an empty tree and iteratively adding each interface into the tree by recursively descending the tree to locate the most suitable leaf node. As a result, complete M-Tree contains each interface as a leaf node. The internal nodes of M-tree contain the routing objects that describe branch objects covering radius, and distances to each child node where the distance is defined by our feature-based similarity measure. To search for a similar interface, one recursively traverses all the paths that satisfy the distance restriction starting from the root. The methodology is applied to the same set of 2,806 interfaces (see previous subsection).

We assess the accuracy of each interface query by finding if the retrieved similar interface has the lowest value of *iiRMSD* among all interfaces in the data set. Specifically we introduce a retrieval error, *E_R_*:
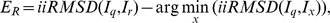
where *I_q_* is a query interface, and *I_r_* is a retrieval interface. The efficiency of each method will be estimated by the average retrieval time.

## Results

In this section, we first present the results of comparing two structure-based similarity measures. Second, we describe evaluation results for a new feature-based similarity measure. Third, we compare our similarity-based classification with the currently existing methods. Then, we introduce the proof-of-concept of a hierarchical classification system for similar protein-protein interactions. We conclude with the description of several case studies of similar interfaces.

### Comparison of structure-based interface similarity measures

To compare accuracies of the two structure-based similarity measures, *iiRMSD* and *siRMSD*, we applied them to a set of 5,924 similar and 4,005 dissimilar pairs of protein interfaces (see section *Evaluating structure-based similarity measures* under *Methods*). The interfaces were obtained from 2,816 protein complexes sampled from the 3Dcomplex dataset [19]. In total, 8,614 binary interaction interfaces formed by 9,144 subunits were extracted from these complexes, averaging ∼3 binary interfaces per complex. Among 8,614 binary complexes, 81.8% were homodimers, and 18.2% were heterodimers.

**Figure 4:**
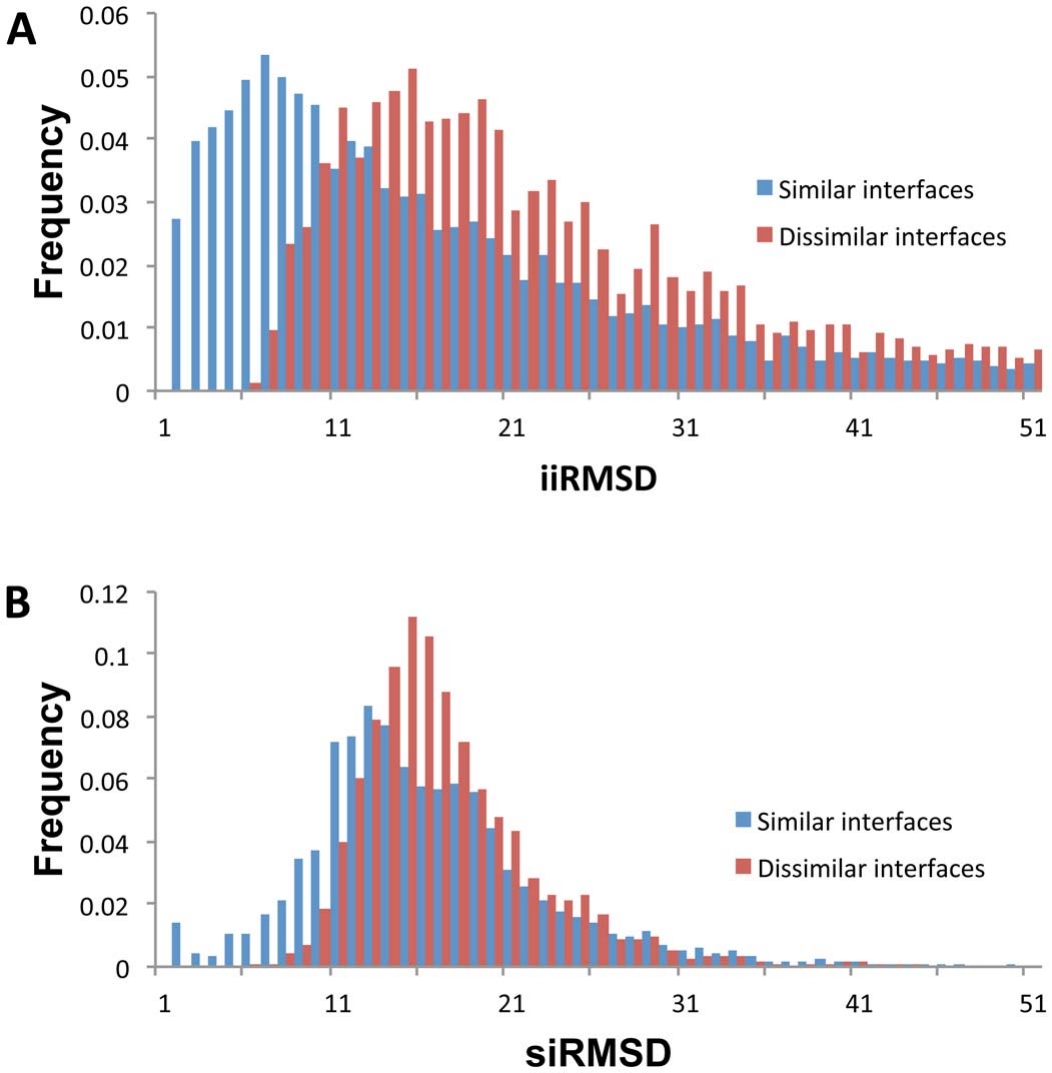
Histograms of the distributions of (A) *iiRMSD* and (B) *siRMSD* values on the datasets of similar and dissimilar interfaces. Both datasets are obtained from 3D Complex database. On average, the dissimilar interface pairs had larger *iiRMSD* and *siRMSD* values (mean values are 20.6 and 15.8, correspondingly) than similar pairs (mean values are 14.8 and 14.7). In addition, the mean value difference between the similar and dissimilar interfaces was larger when using the *iiRMSD* measure (*Δμ* is 4.7 for *iiRMSD* and 1.1 for *siRMSD*).

The analysis of the *iiRMSD* and *siRMSD* value distributions for the similar and dissimilar interfaces (Fig. 4) revealed that on average, the dissimilar interface pairs had larger *iiRMSD* and *siRMSD* values (mean values are 20.6 and 15.8, correspondingly) than similar pairs (mean values are 14.8 and 14.7). In addition, the mean value difference between the similar and dissimilar interfaces was larger when using the *iiRMSD* measure (*Δμ* is 4.7 for *iiRMSD* and 1.1 for *siRMSD*). A more detailed analysis using Bhattacharyya Coefficient based metric [20] also showed the larger distance between the distributions of *iiRMSD* values for the similar and dissimilar interface pairs (*d_BC_* = 0.36), compared to the distributions of *siRMSD* values (*d_BC_* = 0.23). This suggests that *iiRMSD* may differentiate better between the similar and dissimilar interfaces than *siRMSD*. Therefore, for our SVM-based approach, we used *iiRMSD* to select similar and dissimilar interfaces for the training sets.

### Assessment of the new feature-based interface similarity measure

**Figure 5:**
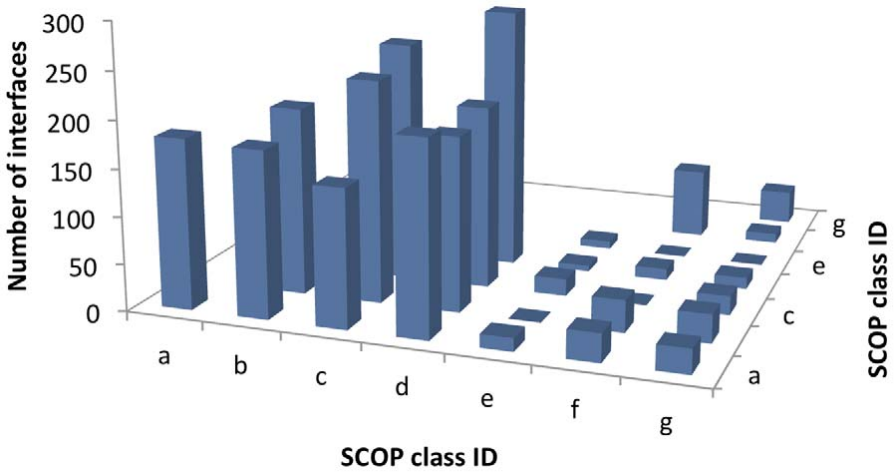
Distribution of SCOP class ID pairs from the training dataset of protein-protein interactions. The dataset covers all SCOP class IDs, while the uneven distribution of the pairs is consistent with the unevenness in the overall distribution of protein structures across the SCOP classes.

We first obtained a set of positive examples consisting of 852 similar interface pairs and a set of negative examples consisting of 1,322 dissimilar interface pairs (Table 1). Both positive and negative datasets were scattered across all major SCOP classes (SCOP class IDs are from *a* to *g*). The majority of interactions, however, were mediated by the subunits from four SCOP classes, *a*, *b*, *c*, and *d* (Fig. 5), which was consistent with the unevenness of the protein structure distribution across the SCOP classes (SCOP release version 1.75, June 2009 [24]).

The leave-one-out cross-validation was done for each SVM model using the same positive set and two different negative sets (*Negative_ND_* for *Model_ND_*, and *Negative_ND_* and *Negative_NN_* for *Model_NDNN_*). For each model, we tested both kernels, polynomial and RBF (Table 3). We found that the overall performance of *Model_ND_* (in terms of accuracy, precision, and recall) is significantly better for both kernels than that one of *Model_NDNN_*. A more detailed analysis revealed that the difference was mainly due to a higher rate of the true positives (93.7% for *Model_ND_ vs.* 64.2% for *Model_NDNN_*); the rate of the true negatives was also higher for *Model_ND_* (91.0% for *Model_ND_ vs.* 85.9% for *Model_NDNN_*). *Model_ND_* was also evaluated on a negative set of native-native dissimilar interfaces (*Negative_NN_*) and compared with the leave-one-out evaluation of *Model_NDNN_* on the same set. We found that being trained on the negative set of native-decoy interface pairs (*Negative_ND_*), *Model_ND_* cannot generalize well to classify dissimilar native-native interface pairs. It was able to correctly classify only 18.5% of the native-native interface pairs; *Model_NDNN_* identified 76.6%, which was similar to its performance on the native-decoy set. Comparing polynomial and RBF kernels revealed similar performances, although the overall performance of the RBF kernel was slightly better for both SVM models. Finally, we found that the performance of both similarity measures was several percent better when considering a positive set consisting exclusively of the interfaces at H-level, compared with the positive set consisting of the interfaces at C-level. For instance, the cross-validation accuracy when using RBF kernel and testing both models on similar interfaces at H-level was 92.8% for *Model_ND_ and* 84.5% for *Model_NDNN_*. Similarly, the cross-validation accuracy, using the same kernel, while testing both models using similar interfaces at C-level was 90.5% for *Model_ND_* and 77.0% for *Model_NDNN_*.

**Table 3:** Leave-one-out cross validation of two SVM models.

	*Model_ND_*			*Model_NDNN_*		
Kernel	Acc	Pre	Rec	Acc	Pre	Rec
RBF	92.6%	93.7%	93.7%	77.4%	74.6%	64.1%
Polynomial	92.0%	92.8%	93.7%	76.5%	70.1%	69.6%

*Model_ND_* is trained on *Positive_H_*, *Positive_C_*, and *Negative_ND_*. *Model_NDNN_* is trained using the same positive set, and a negative set that includes *Negative_ND_* together with *Negative_NN_*. Accuracy (Acc), precision (Pre), and recall (Rec) were calculated for both kernerls, RBF and Polynomial.

The 106 features may not have equal contributions to the feature-based similarity measure (Table 4). The evaluation of features using Weka identified the most important features for both models (Table 4 and Table 5). While the sets of top 20 ranked features for both models had only 5 features in common, the highest ranked feature, defined as the difference of number of contacts between two interfaces, was the same. Other important common features included planarity and ASA of the first interface, as well as the number of contact pairs in the second interface formed either between the aromatic and hydrophobic or between the negative and hydrophobic residues.

**Table 4:** Top 20 ranked features for both SVM models.

Model No. 1		Model No. 2	
Feature ID	Description of features	Feature ID	Description of features
105	difference of number of contacts between two interfaces	105	difference of number of contacts between two interfaces
29	ASA of first interface	30	planarity of first interface
81	ASA of second interface	64	number of Aromatic-Hydrophobic contacts in the second interface
30	planarity of first interface	76	number of Small-Hydrophobic contacts in the second interface
64	number of Aromatic-Hydrophobic contacts in the second interface	29	ASA of first interface
53	number of Aliphatic-Aliphatic contacts in the second interface	83	protrusion of the second interface
71	number of Negative-Negative contacts in the second interface	21	number of Negative-Hydrophobic contacts in the first interface
82	planarity of second interface	44	ratio of Asn hotspots in the first interface
28	number of Polar-Polar contacts in the first interface	16	number of Positive-Small contacts in the first interface
69	number of Positive-Hydrophobic contacts in the second interface	34	ratio of Cys hotspots in the first interface
86	ratio of Cys hostspots in the second interface	50	ratio of Ile hotspots in the first interface
92	ratio of Phe hotspots in the second interface	73	number of Negative-Hydrophobic contacts in the second interface
90	ratio of Tyr hotspots in the second interface	106	difference of ASA between two interfaces
73	number of Negative-Hydrophobic contacts in the second interface	19	number of Negative-Negative contacts in the second interface
74	number of Negative-Polar contacts in the second interface	11	number of Aromatic-Small contacts in the second interface
62	number of Aromatic-Negative contacts in the second interface	100	ratio of Thr hotspots in the second interface
67	number of Positive-Negative contacts in the second interface	68	number of Positive-Small contacts in the second interface
58	number of Aliphatic-Hydrophobic contacts in the second interface	98	ratio of Glu hotspots in the second interface
97	ratio of Lys hotspots in the second interface	39	ratio of Gln hotspots in the first interface
56	number of Aliphatic-Negative contacts in the second interface	33	ratio of Trp hotspots in the first interface

The ranking was obtained using the SVM attribute evaluating protocol implemented in Weka software package.

**Table 5:** Minimum, Maximum, and Median of feature values for top 20 ranked features for both SVM models.

Model No. 1							Model No. 2						
	Positive set			Negative set				Positive set			Negative set		
ID	Min	Max	Med	Min	Max	Med	ID	Min	Max	Med	Min	Max	Med
105	0.00	328.00	35.00	2.00	732.00	130.00	105	0.00	328.00	35.00	0.00	732.00	103.00
29	35.40	146.80	51.20	31.90	168.40	69.90	30	1.48	8.16	4.59	0.48	12.90	4.15
81	0.00	0.23	0.11	0.05	0.19	0.11	64	0.00	0.14	0.03	0.00	0.37	0.02
30	0.00	0.36	0.09	0.00	1.00	0.13	76	0.00	0.50	0.08	0.00	0.20	0.08
64	0.00	0.14	0.03	0.00	0.09	0.02	29	35.30	146.80	51.10	31.90	168.40	61.30
53	0.00	0.09	0.01	0.00	0.12	0.01	83	0.00	55.40	4.49	0.00	55.40	4.49
71	0.00	0.09	0.01	0.00	0.04	0.01	21	0.00	0.09	0.01	0.00	0.33	0.02
82	1.08	9.03	4.52	3.60	8.45	5.13	44	0.00	0.33	0.04	0.00	1.00	0.03
28	0.00	0.36	0.09	0.00	1.00	0.13	16	0.00	0.15	0.02	0.00	0.30	0.02
69	0.00	0.08	0.01	0.00	0.05	0.01	34	0.00	0.27	0.00	0.00	0.33	0.00
86	0.00	0.27	0.00	0.00	0.19	0.00	50	0.00	0.33	0.06	0.00	1.00	0.04
ß92	0.00	0.37	0.05	0.00	0.18	0.04	73	0.00	0.17	0.01	0.00	0.28	0.02
90	0.00	1.00	0.07	0.00	0.31	0.07	106	0.01	84.40	6.37	0.01	122.10	17.90
73	0.00	0.17	0.01	0.00	0.09	0.02	19	0.00	0.05	0.00	0.00	0.12	0.00
74	0.00	0.16	0.02	0.00	0.09	0.02	11	0.00	0.17	0.02	0.00	0.20	0.01
62	0.00	0.07	0.00	0.00	0.04	0.01	100	0.00	0.33	0.06	0.00	0.50	0.06
67	0.00	0.08	0.01	0.00	0.05	0.01	68	0.00	0.19	0.02	0.00	0.22	0.02
58	0.00	0.27	0.04	0.00	0.14	0.03	98	0.00	0.55	0.06	0.00	0.50	0.0ß7
97	0.00	0.33	0.05	0.00	0.30	0.08	39	0.00	0.28	0.04	0.00	1.00	0.03
56	0.00	0.07	0.00	0.00	0.05	0.01	33	0.00	0.25	0.00	0.00	0.50	0.00

For each of the top 20 ranked features (ID stands for the feature ID), the minimum (Min), maximum (Max), and median (Med) values were individually calculated for the positive and negative sets.

### Comparison to existing interface classification methods

To further evaluate the obtained SVM interface similarity classifiers, each classifier was compared to the state-of-art methods to classify protein-protein interfaces, SCOPPI [16] and Prism [17]. For both methods, the similarity of the interfaces was defined through their classification. Two interfaces were defined similar/dissimilar if they belonged to the same/different SCOPPI or Prism class, respectively. The classification data included 8,205 clusters of similar interfaces for Prism and 10,269 clusters for SCOPPI; they were provided by the research groups who developed the methods. We first tested both methods on the positive subset of the training set (Table 6). Since in the provided SCOPPI and Prism datasets, the classification was done exclusively to the sets of similar interactions, we only considered a subset of the positive set that included interaction pairs at H-level. We found that SCOPPI correctly classified 48.0% and PRISM only 15.9% of homologous interfaces from our training set. Such performance could be attributed either to a limited coverage of the classification systems or to a low accuracy of the similarity measures. In comparison, *Model_ND_* correctly predicts the homologous interfaces in 98.1%, while *Model_NDNN_* does so in 75.0% (based on the leave-one-out cross-validation results for homologous interfaces).

**Table 6:** Comparison of SCOPPI, PRISM with *Model_ND_* and *Model_NDNN_*.

Dataset	Classified	SCOPPI	Prism	*Model_ND_*	*Model_NDNN_*
H-level	Similar	48.0%	15.9%	98.1%	75.0%
	Dissimilar	51.0%	3.2%	1.88%	25.0%
	Unknown	1.0%	80.9%	0.0%	0.0%
Dissimilar native-native	Similar	0.0%	0.0%	-	33.6%
	Dissimilar	98.1%	6.6%	-	66.4%
	Unknown	1.9%	93.4%	-	0.0%

The accuracies for each classifier were calculated using homologous interfaces from the positive set and dissimilar native-native interfaces from the negative sets. The results for *Model_ND_* and *Model_NDNN_* were based on the leave-one-out cross-validation. Unknown classification results refer to the percentage of those interface pairs that were not classified by either SCOPPI or Prism.

We next tested the two methods on a negative subset of the training set (Table 6). As both classification systems are for comparing two biological interactions, we excluded the decoy-native interface pairs from the negative set. We found that SCOPPI was able to correctly detect 98.1% of dissimilar pairs and Prism did so for only 6.6%, with the remaining 93.4% of pairs being unclassified. We compared the results only with *Model_NDNN_*, which correctly classified 66.4% of dissimilar interfaces. *Model_ND_* was trained to distinguish only between the decoy and native interfaces, and thus performed poorly on the dissimilar native-native interface pairs.

### Hierarchical classification of similar interactions

**Figure 6:**
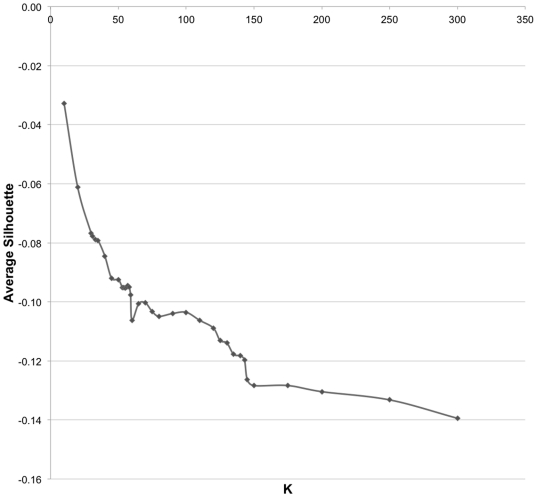
Average Silhouette value against different number of clusters (K). An obvious knee point (K = 140) is selected as the number of clusters.

Our next goal was to construct a proof-of-concept of a biologically meaningful classification of the interaction interfaces, using the feature-based similarity measure. For this purpose, we used the second SVM model, due to its consistency on both positive and negative datasets of the native-native interfaces. The similarity measure was used to obtain the all-against-all SVM distance matrix for the set of 2,806×2,806 interfaces. The cluster analysis using Silhouette method resulted in the number of clusters *K* = 140, which were the clusters at A-level (Fig. 6). Following the protocol to cluster the interfaces at the other two levels (see section *A machine learning approach* in *Methods*), we obtained 1,892 clusters at C-level, and 2,085 clusters at H-level (Table 7). Out of 2,806 randomly sampled interactions, 1,610 and 1,363 interactions formed 1-member clusters at the H-level and C-level, respectively. The overall clustering procedure took 71 hours and 18 minutes on a single core of the Intel Xeon Quad processor (2.4 GHz). The current bottleneck is the feature calculation, which took 70 hours and 9 minutes. Calculating the SVM-based similarity took 30 minutes and hierarchical clustering took another 30 minutes. The theoretical time complexities for each of the three steps are O(*N*), O(*N* ^2^), and O(*N* ^2^), where *N* is the number of interfaces.

**Table 7:** A three-level hierarchy obtained using the new feature-based interface similarity measure.

Level	Clusters	Avg	Min	Max	1-member
H	2,085	1.4	1	9	1,610
C	1,892	1.5	1	13	1,363
A	140	20.0	3	83	0

For each level, the number of clusters (Clusters), the average, minimum, and maximum numbers of members per cluster (Avg, Min, and Max), and the number of clusters with one member (1-member) were calculated.

### Evaluation of the interaction interface retrieval

We next assess the performance of the feature-based similarity measure in the search and retrieval of an interface from a large interface dataset. We first randomly selected 100 interfaces from the whole dataset and used each interface as a query. The remaining 2,706 protein interfaces were used to build an M-Tree (see subsection *Similarity-based retrieval of interaction interfaces* under *Methods*). We calculated the average retrieval error *E_R_^AVE^* and results showed that for 20% of queries, *E_R_^AVE^*<0.28 Å, for 50% of queries *E_R_^AVE^*<1.25 Å, and for 80% of queries *E_R_^AVE^*<3.8 Å. The average retrieval time was 0.8 s. The experiments were conducted on a Linux server with AMD Opteron dual-core 1000 series processors and 2GB RAM.

### Homology and analogy in protein-protein interactions: Case studies

Using results of the hierarchical clustering, a detailed case study analysis was performed. For this analysis, we considered pairs of protein complexes with detected interface similarity at each of the three levels of hierarchy.

This example allowed us to formulate a hypothesis about a new conservation mechanism in charged residues located at the interfaces. Indeed, one would expect from two homologous and highly similar interactions to have conserved charged residues, since the latter usually play an important role in forming the protein interactions. However, when comparing the positions of charged between the interfaces, contrary to these expectations, we found the charged residues in different locations. From the point of view of sequence or structure alignment, this would mean that the charged residues are not conserved, yet they are still presented in both interfaces.

**Figure 7:**
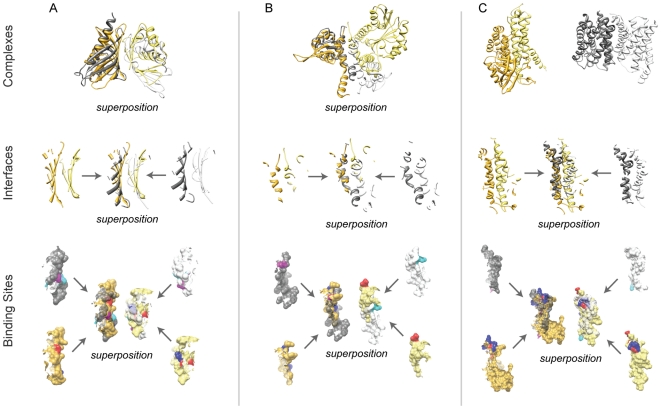
Case studies of similar interactions. (A) H-level interactions (*iiRMSD* = 2.93 Å), (B) C-level interactions (*iiRMSD* = 6.12 Å), and (C) A-level interactions (*iiRMSD* = 6.19 Å). Subunits from the first interaction together with the corresponding interface and binding sites are colored gold and light yellow. Subunits from the second interaction (and their interfaces and binding sites) are colored dark and light grey. Positively and negatively charged residues in the first interaction are colored blue and red, while in the second interaction they are colored cyan and magenta, correspondingly. Superposition refers to the superposed interactions, interfaces, and binding sites.

In the first case study (Fig. 7A), the interfaces clustered at H-level are both formed by homodimers whose subunits belong to the same SCOP Superfamily (SCOP ID: 54427). The first interface is formed by two nuclear transport factor-2 subunits (PDB ID: 1gyb, chains C, D), and the second interface by the association domains of Ca(2+)/calmodulin-dependent protein kinase II (PDB ID 1hkx, chains I, J). While subunits from each interaction belong to a different SCOP Family (SCOP IDs are 54431 and 89851 for subunits forming the first and second interactions, correspondingly), structural superposition of the interfaces revealed their significant structural similarity (here and further, the interface superposition was done by MAPPIS software [44]). We next analyzed the conservation of charged residues between the interfaces. The first interface had two pairs of charged interacting residues. Since charged residues often play an important role in the protein interactions, we expected that the charged residues in the two homologous and highly similar interactions were structurally and sequentially conserved. On the contrary, we detected seven charged residue pairs in the second interface. When the corresponding binding sites were superposed, we found that that these charged residue pairs are not structurally conserved between the two interfaces.

For our next case study (Fig. 7B), we selected two interfaces clustered into the same C-level cluster. One interface is formed by an intra-chain interaction between the N- and C-terminal domains of O-methyltransferase (PDB ID: 1kyw, chain A), while another is formed by an inter-chain interaction between two C-terminal domains of another O-methyltransferase homodimer (PDB ID: 1tw2, chains A and B). Since N- and C- terminal domains of the two O-methyltransferases are not structurally related, the two interactions are not homologous. The complexes were then superposed by aligning the only two structurally similar subunits. Surprisingly, we found that (i) binding sites forming the two interfaces have geometrically similar surfaces, and (ii) locations of the binding sites on the surfaces of structurally similar subunits are in close proximity and are partially overlapped. Moreover, when analyzing the conservation of the charged residues in these interfaces, we observed an intriguing phenomenon. We detected a pair of charged residues whose location was conserved between the two interfaces but whose charges were swapped when comparing one interface with another (LYS 117.A in contact with ASP 120.A in the first interface, and GLU 89.A in contact with ARG 17.B in the second one).

Finally, in the third case study (Fig. 7C), we considered two structurally unrelated binary complexes that were clustered into the same A-level cluster. The first complex is an intra-chain interaction of the C- and NM- domains of acyl-CoA dehydrogenase (PDB ID: 1ege, chain C) and the second one is a glycerol-conducting channel homodimer (PDB ID: 1fx8, chain A). The subunits for the two complexes were from structurally unrelated SCOP Superfamilies (SCOP IDs are 47203 and 56645 for the first complex, and 81338 for both subunits of the second complex). The analysis of the interfaces showed their significant similarity in shape and secondary structure. However, the interface in the first complex had multiple charged residues agglomerated at one part of the interface, while the interface of second complex had a single pair of the charged residues. In addition, the analysis of the charged residues revealed that they were located on the opposite sides of the two interfaces.

## Discussion

In this paper, we present an accurate alignment-free interface similarity measure and demonstrate its advantages and applicability. We have shown that the measure has a significantly greater coverage than the alignment based methods while preserving high accuracy. In addition, we have demonstrated that the high coverage allows generating a comprehensive SCOP-like hierarchical classification of similar interaction interfaces as well as efficiently solving the interface search and retrieval problem. Finally, we have presented an example of how the measure could be used to suggest a new biological phenomenon.

Throughout this work, we have constructed three datasets of interaction interfaces. The first dataset consists of (i) homologous interface pairs that are obtained exclusively from structurally similar binary complexes extracted from 3D Complex database, and (ii) dissimilar interface pairs obtained from the same database. The purpose of this dataset is determining which of the structure-based similarity measures is more accurate: the one that relies on superposition of the entire subunits, or the one that relies on the interaction interfaces only. In the second dataset, we collected as diverse datasets of similar and dissimilar interfaces as we could reliably get using a structure-based similarity measure. Our protocol removes potential bias in the interaction data, by ensuring that each family of structurally similar subunits contributes equally to the dataset. While this is an important step for an accurate SVM training, the protocol would not reflect the actual distribution of the interactions across the pairs of homologous families. To account for that, we built the third dataset, which not only serves as a test bed for constructing a classification system of the entire structural interactome, but also allows us to study biological phenomena occurring in similar interfaces. All datasets can be downloaded at: http://korkinlab.org/datasets/i_similarity/i_sim_data.html

Based on the assessment results of the two SVM classifiers and their comparison with the state-of-art interface classification systems, we have made several conclusions. First, we suggest that the *Model_ND_* can be efficiently used when modeling protein-protein interactions by a comparative approach, *e.g.*, comparative docking, where the modeled interfaces are matched against a database of biological interfaces. Second, we conclude that the main advantages of our approach, compared to the current methods, include better coverage and higher accuracy on detecting similar interfaces. On the other hand, our approach could further benefit from improving the detection of dissimilar interfaces.

Hierarchical classification of the interaction interfaces resulted in a significant number of 1-member clusters at C- and H-levels. This is not surprising, as the interfaces clustered into the same C- or H-level cluster have an additional constraint: one or both interacting subunits must belong to the same SCOP superfamily. The probability of two interactions to have one of the two subunits in the same SCOP superfamily is small, since the average number of members per each SCOP superfamily in the considered set of non-redundant interactions (∼2.3) is significantly smaller than the total number of SCOP Superfamilies for the same set (1,225). As a result, the total number of expected clusters with multiple interactions is expected to be low at C- and H-levels.

The performance analysis of the hierarchical classification protocol suggests that expanding the hierarchical classification to the entire set of protein-protein interactions is feasible. Indeed, the feature calculation, while taking the most time per each interface among the three steps (see section *Hierarchical classification of similar interactions* under *Results*), has the complexity that is linear of the number of available binary interactions. Thus, since the current dataset constitutes ∼1% of the structural interactome [39] this step can be completed in the same time (∼70 hrs) but on a 100-node cluster. Due to their quadratic complexities, stages two and three are expected to take ∼50 hrs each on the same cluster.

We have also demonstrated the applicability of the feature-based similarity to the problem of interface search and retrieval. Specifically, for a query interface one can accurately and efficiently find a similar interface from a large interface dataset. This proof-of-concept may have important implications for other bioinformatics approaches, *e.g.* for comparative docking, where the candidate interface models are searched against the database of native interfaces or for functional annotation of novel protein interactions.

Finally, for each case study, we have detected and analyzed the charged residues located at the interfaces. The analysis has revealed an interesting phenomenon, where the relative positions of charged residues in similar interfaces are either swapped between the interacting binding sites or appear in different regions of the interfaces. The principal role of the charged residues in forming interaction interfaces has been well studied [45], [46], [47]. However, a recent analysis of the residue conservation in the protein interfaces showed that the charged residues are less conserved than hydrophobic or aromatic residues [48]. The properties of the charged residues found in our case studies are consistent with that conclusion. Our findings may also suggest that for some protein-protein interactions, a mere presence of the charged residues in the interface, not requiring the conservation of charged residue locations at the interface, is sufficient to the complex formation.
